# Ornithine Transcarbamylase ArgK Plays a Dual role for the Self-defense of Phaseolotoxin Producing *Pseudomonas syringae* pv. *phaseolicola*

**DOI:** 10.1038/srep12892

**Published:** 2015-08-10

**Authors:** Li chen, Pin li, Zixin deng, Changming zhao

**Affiliations:** 1 Key Laboratory of Combinatorial Biosynthesis and Drug Discovery, Ministry of Education, and School of Pharmaceutical Sciences, Wuhan University, Wuhan, 430071, China.

## Abstract

*Pseudomonas syringae* is a phytopathogenic bacterium widely spread on terrestrial plants. Sulfodiaminophosphinyl tripeptide Phaseolotoxins (PHTs), produced by *P. syringae* pv. *phaseolicola* and *P. syringae* pv. *actinidiae*, represent a kind of antimetabolic phytotoxins. PHTs inhibit host cell Ornithine transcarbamylase (OTCase) activity and induce Arginine auxotrophic phenotype. The biosynthesis of PHT is temperature dependent, being optically produced at around 18 °C, while blocked above 28 °C. PHT resistant OTCase ArgK acts as a functional replacement of housekeeping OTCase ArgF, which is the acting target of PHT, to confer PHT producers with self-resistance. It was postulated that *argK* might be regulated directly by a PHT biosynthetic precursor and indirectly by temperature with an unknown manner. Neither transcriptional regulator nor thermal regulation related protein encoding gene was detected from PHT biosynthetic gene cluster. The tripeptide, Cit-Ala-*h*Arg, was identified to be a by-product of PHT biosynthetic pathway in this report. Formation of Cit-Ala-*h*Arg was catalyzed by ArgK with tripeptide Orn-Ala-*h*Arg and carbamyl phosphate as substrates. It showed that ArgK not only provided alternative Arginine source as reported previously, but also controlled the production of PHTs by converting PHT biosynthetic precursors to nontoxic Cit-Ala-*h*Arg reservoir for producers’ self-defense.

*Pseudomonas syringae* (*P. syringae*) is a group of phytopathogenic bacterial species which include more than 50 pathovars. *P. syringae* spreads on most of the terrestrial plants and induces a wide variety of diseases^1^,^2^. Phosphorotriamidate natural product Phaseolotoxins (PHTs) producers, *P. syringae* pv. *phaseolicola* and *P. syringae* pv. *actinidiae*, cause halo blight disease on beans and bacterial canker on kiwifruits respectively^3^,^4^,^5^. PHTs [N^δ^(N’-sulfo-diaminophosphinyl)-ornithyl-alanyl-homoarginine] were readily degraded by nonspecific peptidases *in planta* to produce compound N^δ^ (N’-sulfo-diaminophosphinyl)-ornithine (PSOrn)^6^,^7^,^8^. PSOrns are irreversible inhibitor of ornithine transcarbamylase (OTCase) attributed to the chemical structure similarity with tetrahedral intermediates in OTCase mechanism^6^. OTCase catalyzes the formation of Citrulline with Ornithine and carbamyl phosphate as substrates. Inhibition of OTCase blocks the biosynthesis of Arginine *in vivo* and leads to a reduction of protein synthesis^9^. In addition, PHTs are competitive inhibitors to mammalian and bacterial OTCase, including the OTCase ArgF of PHT producing strain *P. syringae* pv. *phaseolicola* 1448A^10^,^11^,^12^. So, PHT producing *Pseudomonas* cells must employ certain approaches to protect themselves from being killed by their own second metabolic products.

PHT resistant OTCase ArgK, encoded by *argK*, is a well-known self-resistance conferring element to PHT producers^13^,^14^,^15^. In *P. syringae* pv. *phaseolicola* cells, ArgK acts as a functional replacement of housekeeping OTCase ArgF to provide an alternative Arginine source whenever ArgF is inhibited by PHTs^3^,^16^. PHT biosynthetic gene cluster contains 23 genes (24.8 kb), which organized into five transcriptional units, two monocistronic units (*argK* and *phtL)* and three operons^17^,^18^,^19^. Biosynthesis of PHT is temperature dependent, being optimized between 18 °C and 20 °C, while blocked above 28 °C^20^,^21^. Resistant gene *argK* expressed at both 18 °C and 28 °C. However its transcriptional level was much lower at 28 °C^17^. It was postulated that *argK* might be regulated directly by a PHT biosynthetic precursor and indirectly by temperature. A protein-binding DNA motif has been found out in *argK* promoter region, while no repressor protein involved in thermoregulation of PHT biosynthesis has been identified so far^22^,^23^,^24^.

Gene *phtL* expressed at both 18 °C and 28 °C, and showed no quantity difference at transcriptional level^17^. PhtL, the product of *phtL*, is a bidomain enzyme which shows distantly similarity to pyruvate phosphate dikinase (PPDK) and phosphoenolpyruvate synthase (PS). It has been proposed that *phtL* played a regulatory function in PHT biosynthesis^17^,^25^. The amidinotransferase homolog encoded by *amtA*, was proposed to catalyze the conversion of Lysine to homoarginine (*h*Arg) using Arginine as guanidyl group donors^26^. Two ATP grasp family peptide ligase encoding genes, *phtQ* and *phtU* were recognized and deduced to catalyze the formation of amido bonds of PHT peptide scaffolds Orn-Ala-*h*Arg^27^. *N*-Sulfodiaminophosphinyl groups attach to the delta amino group of Ornithine residues give the chemical structure of PHTs. The chronological order of individual steps involved in PHT biosynthesis and the three Nitrogen-Phosphorus bonds (N-P bonds) biosynthetic mechanism remain poorly understood.

Here, precursor ion scan (PIS) mass spectrometry, feeding experiments and enzyme assay were employed in the identification of PHT biosynthetic pathway by-products, tripeptides Cit-Ala-*h*Arg, which indicating a dual role of ArgK for PHT producers self-defense.

## Results

### PIS mass spectrometry to screen for PHT biosynthetic intermediates

**Figure 1:**
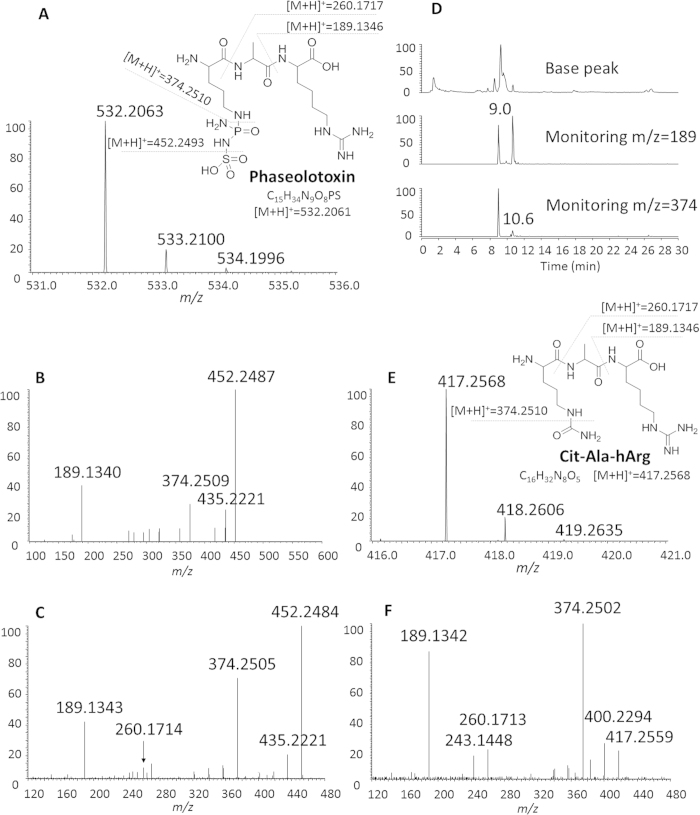
PIS mass spectrometric analysis to screen for PHT biosynthetic intermediates. (**A)** high resolution mass spectrum of PHTs (inset is fragmentation pattern of PHT); (**B**) tandem mass (MS^2^) spectrum of PHTs; (**C**) tandem mass (MS^3^) spectrum of daughter ions with *m/z* ratio at 452.3 in panel (**B**); (**D**) chromatogram of PIS mass spectrometric analysis; (**E**) high resolution mass spectrum of novel ions with *m/z* ratio at 417.2568 (inset is fragmentation pattern of Cit-Ala-*h*Arg); (**F**), tandem mass spectrum of ions with *m/z* ratio at 417.2568.

PHTs were detected in the cultural supernatants of wild type strain *P. syringae* pv. *phaseolicola* 1448A by High resolution mass spectrometry (HR MS) and ^31^P nuclear magnetic resonance (NMR) analysis. The ^31^P chemical shift of PHTs was 10.94 *ppm* (Figure S1). Tandem mass spectra (MS2) indicated that species with *m/z* ratio at 452, 374 and 189 are the characteristic daughter ions of PHTs (*m/z* ratio at 532) (Fig. 1B). Daughter ions with *m/z* ratio at 374 and 189 were also observed in the MS3 spectra when desulfonated PHTs (*m/z* ratio at 452) were further fragmented (Fig. 1C).

PHTs characteristic daughter ions with *m/z* ratio at 374 and 189 were used as queries to screen for PHT biosynthetic intermediates by PIS mass spectrometry. Novel ion with *m/z* ratio at 417 was detected and tandem mass spectra were further recorded on HR-FT-MS instrument (Fig. 1D,E). Ions with *m/z* ratio at 417, desulfonated PHTs and PHTs shared two identical daughter ions, *m/z* ratio at 374 and 189 (Fig. 1F), which are corresponding to the exact mass of tripeptides Orn-Ala-*h*Arg and *h*Arg residues respectively. It indicated that the novel ion with *m/z* ratio at 417 is a derivate of PHT peptide scaffold Orn-Ala-*h*Arg. Based on exact mass of 417.2568 (Fig. 1E), the side functional group was deduced to be carbamoyl. Carbamylation of Orn-Ala-*h*Arg produced Cit-Ala-*h*Arg, which was a deduced chemical structure of the newly detected ions with *m/z* ratio at 417.

### Tripeptides Cit-Ala-*h*Arg were by-products of PHT biosynthetic pathway

**Figure 2:**
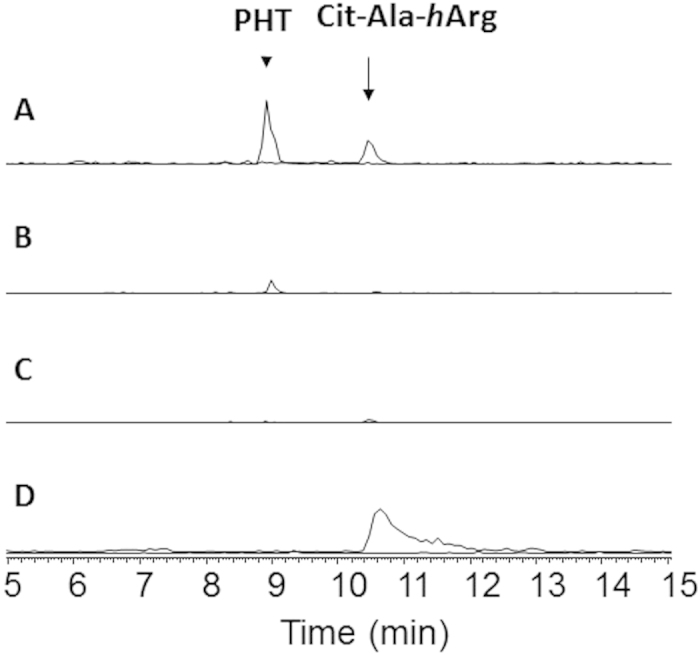
Extracted ion chromatograms of compounds from culture supernatants of PHT producers and gene in-frame deletion mutants. (**A**) 1448A (PHT producer, wild type); (**B**) *phtU*^-^ mutant strain CL001 fed with Orn-Ala-*h*Arg; (**C**) *phtQ*^-^ mutant strain CL002 fed with Orn-Ala-*h*Arg; (**D**) *phtL*^-^ mutant. Upper lines were extracted for PHTs and lower lines were extracted for tripeptides Cit-Ala-*h*Arg, with a tolerance of 0.5 Da.

**Figure 3:**
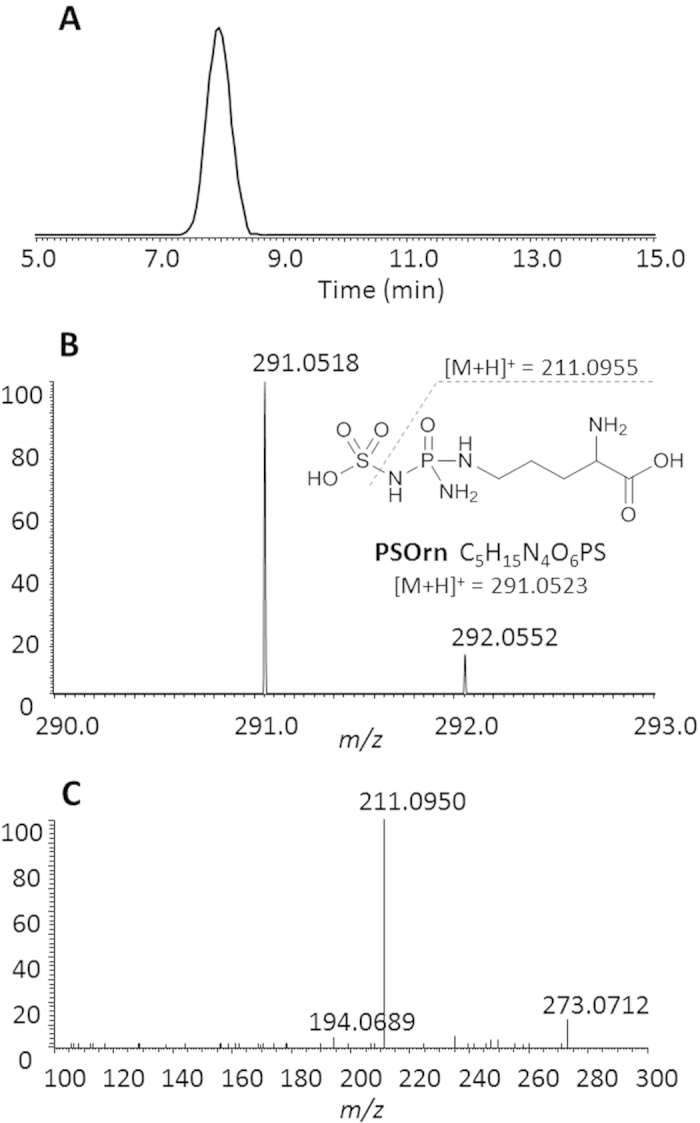
HR MS spectra of PSOrn. (**A**) Extracted ion chromatogram of PSOrn, with a tolerance of 5 *ppm*. (**B**) high resolution mass spectrum of PSOrn (inset is fragmentation pattern of PSOrn); (**C**) tandem mass (MS^2^) spectrum of PSOrn.

To determine the biosynthetic relationship between tripeptides Cit-Ala-*h*Arg and PHTs, *P. syringae* pv. *phaseolicola* 1448A genes *phtU*, *phtQ* and *phtL* were in-frame deleted (Figure S2). Neither Cit-Ala-*h*Arg nor PHT was detected from the culture supernatants of *phtU*^-^ mutant CL001 strain. Gene *phtU* complementation strain CL004 regained the ability to produce PHTs (Figure S3). Authentic _L_-Orn- _L_-Ala- _L_-*h*Arg standards feeding restored *phtU*^-^ mutants with the ability to produce PHTs at 18 °C, which is the temperature permissive for PHTs synthesis in wild type producers (Fig. 2B). Simultaneously, feeding of _L_-Cit- _L_-Ala- _L_-*h*Arg and _L_-Ala- _L_-*h*Arg restored *phtU*^-^ mutants with the ability to produce PHTs as well (Figure S3). To the *phtQ* in-frame deletion, neither Cit-Ala-*h*Arg nor PHT was detected from the culture supernatants of *phtQ*^-^ mutant strain CL002. Surprisingly, PSOrn was detected from the culture supernatants of *phtQ*^-^ mutants. HR tandem MS spectra of PSOrn were shown in Fig. 3. None of the authentic oligopeptides described here restored *phtQ*^-^ mutants with the ability to produce PHTs (Fig. 2C). Meanwhile, gene *phtQ* complementation strain CL005 regained the ability to produce PHTs (Figure S3). Gene *phtL* knockout did not abolish *phtL*^-^ mutant strain CL003 the ability to produce tripeptides Cit-Ala-*h*Arg at 18 °C (Fig. 2D). PhtL is necessary for PHTs biosynthesis since *phtL*^-^ mutants did not produce any PHT. The accumulated amounts of Cit-Ala-*h*Arg were increased when PHT biosynthesis pathway was blocked in *P. syringae* pv. *phaseolicola phtL*^-^ mutants (Fig. 2D).

**Figure 4:**
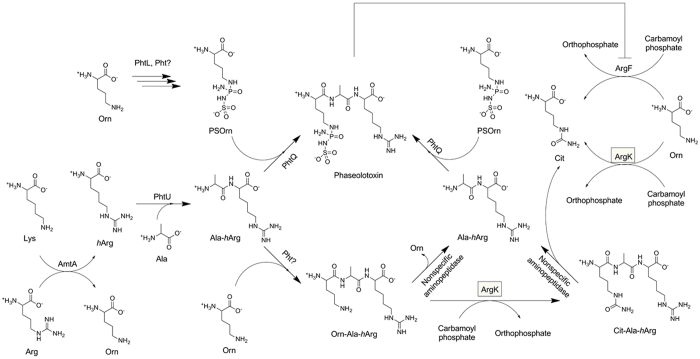
Dual roles of ArgK in PHT producers self-defense. ArgK provided alternative Arginine source by acting as a functional replacement of housekeeping OTCase ArgF, and also controlled the production of PHT by converting PHT biosynthetic precursors to nontoxic Cit-Ala-*h*Arg reservoir.

Gene deletion and feeding experiments results described here showed that tripeptides Cit-Ala-*h*Arg were by-products accumulated during PHTs biosynthetic process. Dipeptide _L_-Ala- _L_-*h*Arg was a joint precursor for the synthesis of both PHT and Cit-Ala-*h*Arg (Fig. 4). Nonspecific aminopeptidase catalyzed hydrolysis of _L_-Orn- _L_-Ala- _L_-*h*Arg and _L_-Cit- _L_-Ala- _L_-*h*Arg would release _L_-Ala- _L_-*h*Arg. Incorporation of _L_-Ala- _L_-*h*Arg into PHT biosynthetic pathway restored *phtU*^-^ mutants with PHTs producing ability (Fig. 4). Detection of precursor PSOrn from the culture supernatant of *phtQ*^-^ showed that PhtQ involved in the biosynthesis of PHTs with PSOrn and _L_-Ala- _L_-*h*Arg as substrates. Accumulation of by-products Orn-Ala-*h*Arg and Cit-Ala-*h*Arg from the culture supernatants of wild type strains and certain gene in frame deletion mutants might be attributed to the substrate tolerance of PhtQ. We can not rule out the possibility that there are certain uncharacterized peptide ligases participated in the formation of Orn-Ala-*h*Arg and/or Cit-Ala-*h*Arg. PhtL might be involved in the biosynthetic steps of PSOrn with _L_-Orn as a close precursor. Based on the fact that there is a *phtL* gene homologous (*agnD1* and *agnD2*) in the biosynthetic gene cluster of another phosphoramidate natural product Agrocin 84^28^,^29^,^30^, we assume that *phtL* is related to the N-P bonds formation of PHTs.

### ArgK catalyzed the formation of Tripeptides Cit-Ala-*h*Arg

To extend our understanding about why such a great amount of by-products were accumulated, it is necessary to address the question concerning formation of tripeptides Cit-Ala-*h*Arg. Two possible mechanisms could be involved to account for this phenomenon. First, PhtQ enzyme is a substrate tolerant peptide ligase and capable of catalyzing the synthesis of Cit-Ala-*h*Arg with Citrulline and dipeptide Ala-*h*Arg as substrates. Second, a potential transcarbamylase catalyzes the carbamylation of Orn-Ala-*h*Arg to form Cit-Ala-*h*Arg. To the PHTs producing *Pseudomonas* cells, it suffers from Citrulline shortage since the Citrulline formation reaction *k*_cat_ catalyzed by ArgK reduced to between 1% and 2% of that catalyzed by typical OTCase, such as ArgF^31^. It is not an economical strategy to produce a great amount of by-product Cit-Ala-*h*Arg by consuming limited Citrulline source. In addition, by-products Cit-Ala-*h*Arg were detected from the cultural supernatants of *phtU*^-^ as well as *phtQ*^-^ mutants when Orn-Ala-*h*Arg was added as substrate respectively (Figure S4). Therefore, the OTCase ArgK was suspected to be a main contributor for the formation of by-product Cit-Ala-*h*Arg *in vivo*.

**Figure 5:**
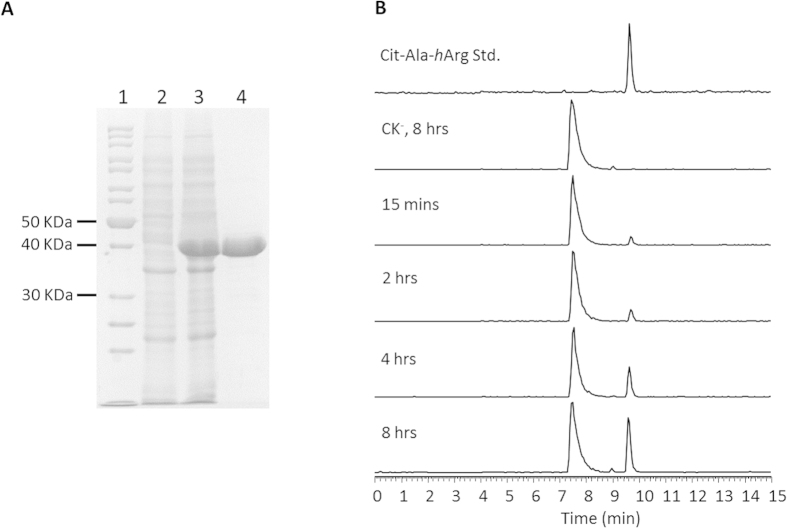
PHT resistant OTCase ArgK enzyme assay. (**A**) SDS-PAGE picture of purified ArgK protein. Lane 1, molecular weight marker; Lane 2, total protein without IPTG inducing; Lane 3, total protein with IPTG inducing; Lane 4, purified ArgK protein. (**B**) Total ion chromatograms of enzyme reaction system analyzed by HPLC-MS with a SIM mass spectrometric method screening *m/z* ratio from 360 to 430. Reaction system was set as 0.2 mg purified ArgK, 20 mM Tris-HCl (pH 8.0), 50 mM NaCl, 1.5 mM Lithium carbamoyphosphate dibasic hydrate, 1 mM _L_-Orn- _L_-Ala- _L_-*h*Arg, 2 mM MgSO_4_ and 0.5 mM ATP, 200 μL in total. CK^-^ means negative control reaction in which ArgK was absent. Peaks of retention time at 7.5 min were substrates _L_-Orn- _L_-Ala- _L_-*h*Arg; peaks of retention time at 9.6 min were products _L_-Cit- _L_-Ala- _L_-*h*Arg.

Enzyme assay showed that purified ArgK catalyzed the formation of _L_-Cit- _L_-Ala- _L_-*h*Arg with _L_-Orn- _L_-Ala- _L_-*h*Arg and carbamyl phosphates as substrates at 28 °C *in vitro* (Fig. 5). The tripeptides _L_-Cit- _L_-Ala- _L_-*h*Arg production was positively correlated with reaction time, while no detectable amount was observed from the negative control reaction in which ArgK was absent (Fig. 5B). On the contrary, ArgK did not catalyze the reverse reaction in which the system was set up with _L_-Cit- _L_-Ala- _L_-*h*Arg and orthophosphate as substrate.

**Figure 6:**
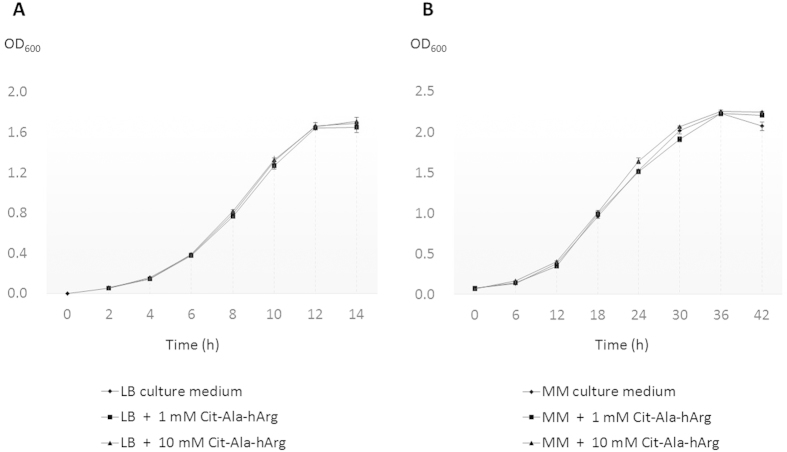
Growth curve of *P*. *syringae* pv. *phaseolicola* 1448A in distinct culture media. (**A**) LB medium with additional 1 mM or 10 mM tripeptide _L_-Cit- _L_-Ala- _L_-*h*Arg. (**B**) MM medium with additional 1 mM or 10 mM _L_-Cit- _L_-Ala- _L_-*h*Arg.

It indicated that ArgK played dual roles for PHT producers’ self-defense, which the first one was providing alternative Arginine source by acting as functional replacement of ArgF as documented previously^13^,^14^,^15^, and the second one was reducing PHTs production by modifying _L_-Orn- _L_-Ala- _L_-*h*Arg to produce by-products _L_-Cit- _L_-Ala- _L_-*h*Arg. Exogenous _L_-Cit- _L_-Ala- _L_-*h*Arg with a concentration range from 1 to 10 mM did not affect the growth of PHTs producer *P. syringae* 1448A (Fig. 6). Tripeptides _L_-Cit- _L_-Ala- _L_-*h*Arg were partially consumed by *P. syringae* cells in the tested conditions.

**Figure 7:**
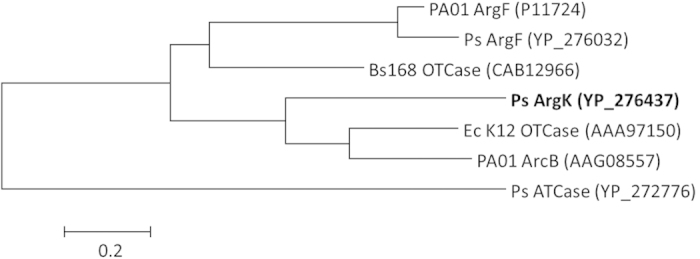
Phylogenetic tree of OTCases analyzed by MEGA6 program. PA01 ArgF, OTCase ArgF of *P*. *aeruginosa* PA01; Ps ArgF, OTCase ArgF of *P*. *syringae* pv. *phaseolicola* 1448A; Bs168 OTCase, OTCase of *Bacillus subtilis* 168; Ps ArgK, PHT resistant OTCase ArgK of *P*. *syringae* pv. *phaseolicola* 1448A; Ec K12 OTCase, OTCase of *Escherichia coli* K-12; PA01 ArcB, catabolic OTCase ArcB of *P*. *aeruginosa* PA01; Ps ATCase, Aspartate carbamyltransferase of *P*. *syringae* pv. *phaseolicola* 1448A. Bracketed is the GenBank accession number.

Besides anabolic OTCase ArgF, another catabolic OTCase ArcB has been detected from *P*. *aeruginosa*^32^,^33^. ArcB is involved in the Arginine deiminase pathway and catalyzes the phosphorolysis of Citrulline to produce Ornithine and carbamyl phosphate^34^,^35^. It is exactly the reverse reaction of that one involved in the Arginine biosynthesis pathway catalyzed by ArgF. Phylogenetic analysis indicated that ArgK is more closely related to ArcB of *P*. *aeruginosa* PA01 than ArgF of *P. syringae* (Fig. 7). Comparing with typical anabolic OTCases, amino acid residue substitutions of ArgK were observed in the conserved sites around ornithine binding “SMG” loop^31^. The newly identified ArgK function announced here accords well with the alteration of substrate binding pocket. PHT resistant and substrate tolerant characters of OTCase ArgK challenge the current paradigm for OTCase.

## Discussion

For pathogens colonization and subsequent symptom development, it is necessary to produce certain amount of virulence factors when *P. syringae* pv. *phaseolicola* cells infiltrated into plant tissues^3^,^4^. Phytotoxic compound PHTs conferred pathogens with survival advantages while burdened themselves with self-toxicity, amino acid source and energy expenditure simultaneously. The productivity and production of PHTs must be controlled accurately. Nevertheless, there is no typical transcriptional regulator gene has been identified from PHT biosynthetic gene cluster so far^4^,^17^.

To the PHT producers, it is a passive strategy to employ PHT resistant ArgK acting as a functional replacement of OTCase ArgF. In this study, we demonstrated that ArgK directly controlled the production of PHTs by carbamylation of Orn-Ala-*h*Arg to produce by-product Cit-Ala-*h*Arg. Cit-Ala-*h*Arg could act as a nontoxic reservoir or be degraded by nonspecific aminopeptidase to provide additional Citrulline source (Fig. 4). It depends on the physiological situation of PHTs producers. This active manner could act as a complementary mechanism to the passive one for PHT producers’ self-defense. The dual roles of ArgK reported here help us to understand how *P. syringae* pv. *phaseolicola* kept a nice balance between amino acid fundamental metabolic pathway and the second metabolites PHTs biosynthesis pathway.

## Materials and Methods

### Fermentation for PHTs producing

Seed cells of *P. syringae* pv. *phaseolicola* strain 1448A and gene in-frame deletion mutants were cultured by LB medium at 28 °C. Cells were harvested and washed twice with equal volume of distilled water, then transferred into sucrose minimal medium and fermented at 18 °C for 72 hours for PHTs producing. In the feeding experiments, 0.5 mM authentic oligopeptides were fed in the fermentation process of *phtU*^-^ and *phtQ*^-^ mutants respectively.

### PHT biosynthesis related genes in-frame deletion and complementation

PHTs native producer *P. syringae* pv. *phaseolicola* strain 1448A genome Fosmid library was constructed with CopyControl™ Fosmid Library Production Kit (Epicentre® Biotechnologies products) according to standard protocols^36^. Fosmid 7C6 with a 33.6 kb DNA insert which covers the complete PHT biosynthetic gene cluster and flanking sequence of 5.5 kb upstream and 3.3 kb downstream was screened out by PCR. Gene *phtU* of Fosmid 7C6 was replaced by Apramycin resistant gene *aac(3)IV* from plasmid pIJ773 by PCR targeting with primer pair DUF1/DUR2 (Table S1)^37^. Gene *aac(3)IV* was eliminated by FLP-recombinase mediated excision in *E.coli* DH5α/BT340 to generate a disruption cassette pWHU2001. Plasmids pWHU2001 were transferred into *P. syringae* pv. *phaseolicola* 1448A cells by electroporation and single crossover recombinant strains resistant to chloramphenicol were picked up. The *phtU* gene in-frame deletion mutant strains CL001 resulted from double crossover were selected out by rounds of relaxation and chloramphenicol sensitivity tests and then validated by PCR with primer pair VLF1/VLR2 (Figure S2A, S2B, Table S1). Identical strategy was employed for gene *phtQ* and *phtL* in-frame deletion. Primer pair DQF1/DQR2 (Table S1) was used in *phtQ* PCR targeting and primer pair VQF1/VQR2 (Table S1) was used in *phtQ*^-^ mutant strain CL002 PCR validation (Figure S2C, S2D). Primer pair DLF1/DLR2 (Table S1) was used in *phtL* PCR targeting and primer pair VLF1/VLR2 (Table S1) was used in *phtL*^-^ mutant strain CL003 PCR validation (Figure S2E, S2F).

To the gene complementation of *phtU* and *phtQ* in CL001 and CL002 strains, the constitutive promotor of gene *phtA* was employed to construct infusion genes P_*phtA*_-ORF_*phtU*_ and P_*phtA*_-ORF_*phtQ*_. A 227 bp DNA fragment containing P_*phtA*_ was amplified by PCR with primer pair P_*phtA*_F1/P_*phtA*_R2 (Table S1). The ORF region of gene *phtU* was amplified by PCR with primer pair PhtUF1/PhtUR2 (Table S1). These two fragments were insert into the *Bam*HI site of a RK2-derived plasmid pRKaraRED to give recombinant plasmid pWHU2004. Transformation of pWHU2004 into *phtU*^-^ mutant strain CL001 gave *phtU* gene complementation strain CL004. Identical strategy was employed for gene *phtQ* complementation strain CL005 construction with the exception of that *phtQ* was amplified with primer pair PhtQF1/PhtQR2 (Table S1) and the recombinant plasmid was defined as pWHU2005.

### ArgK over-expression and enzyme assay

Gene *argK* was amplified by PCR with primer pair OKF1/OKR2 (Table S1) and inserted into the *Bam*HI/*Xho*I locus of vector pET28a. His·tag-ArgK fusion proteins were over-expressed and purified with standard protocol^36^. Authentic standards of _L_-Orn- _L_-Ala- _L_-*h*Arg and _L_-Cit- _L_-Ala- _L_-*h*Arg were purchased from Life Tein L.T.D. Company (Beijing) (Figure S3). Transcarbamylase enzyme assay were performed in 1.5 mL eppendorf tubes with a system as 0.2 mg purified ArgK, 20 mM Tris-HCl (pH 8.0), 50 mM NaCl, 1.5 mM Lithium carbamoyphosphate dibasic hydrate, 1 mM _L_-Orn- _L_-Ala- _L_-*h*Arg, 2 mM MgSO_4_ and 0.5 mM ATP, 200 μL in total. ArgK was absent in the negative control reactions while the other factors remained identical. The enzyme was removed from the reaction system with a 10 KDa centrifugal filter at 10,000 *rpm* for 10 minutes. The residual sections were diluted to ten times volume of the original reaction system before injected into the HPLC-MS analyzer.

### HPLC-MS analysis

Culture supernatants for PHTs and biosynthetic intermediates discovery were thirty times concentrated by rotovaping at room temperature and then cut by methanol with a final concentration of 90%. Samples were dried out by rotovaping and re-suspended in ddH_2_O for HPLC-MS analysis. A Thermo hypercarb column (100 × 2.1 mm, 5 μm, column temperature 30 °C) with a flow rate of 0.4 mL/min was employed in the HPLC section. The HPLC gradient was 0% solvent B (4 min), 0–40% solvent B (12 min), 40–100% solvent B (3 min), 100% solvent B (3 min), 100–0% solvent B (3 min), 0% solvent B (5 min). Solvent A was 0.1% formic acid in H_2_O and solvent B was 0.1% formic acid in CH_3_CN.

High resolution MS analysis was carried out in a positive mode on a Thermo Scientific LTQ XL Orbitrap mass spectrometer as described previously^38^. PHTs samples from the culture supernatants of *P. syringae* pv. *phaseolicola* strain 1448A were used to build the MS fragmentation fingerprint of PHTs and desulfonated PHTs by selected ion reaction (SIR) method. Daughter ions with *m/z* ratio at 189 and 374 were recognized to be the characteristic fragments of PHTs. To screen for PHTs biosynthetic intermediates, PIS was performed in a positive mode on a Thermo Scientific TSQ Quantum Access MAX instrument (monitoring *m/z* ratio at 189 and 374) equipped with a Thermo Scientific Accela 600 pump. Target ions were selected out for HR tandem MS analysis as described above to figure out the elemental compositions.

Enzyme assay samples were analyzed on the TSQ instrument with a selected ion monitoring (SIM) scan mode with a range from *m/z* ratio at 360 to 430. Reaction products were double checked by HR tandem MS analysis on the Orbitrap instrument.

### NMR analysis

^1^H NMR and ^13^C NMR spectra of tripeptides _L_-Orn- _L_-Ala- _L_-*h*Arg (20 mg) and _L_-Cit- _L_-Ala- _L_-*h*Arg (20 mg) were recorded on Agilent 400 MHz instrument in D_2_O, respectively (Figure S3). Culture supernatants of *P. syringae* pv. *phaseolicola* strain 1448A were cut by methanol and dried out as described above. Residues were re-suspended in ddH_2_O with 10% D_2_O and ^31^P NMR spectra were recorded on Agilent 400 MHz instrument.

### Bioassay of *P. syringae* 1448A against _L_-Cit- _L_-Ala- _L_-*h*Arg

To evaluate the toxicity of _L_-Cit- _L_-Ala- _L_-*h*Arg to PHT producers *P. syringae* 1448A, authentic tripeptide L-Cit- L-Ala- L-hArg was added to the culture media of 1448A. Two kinds of media, LB (Tryptone 10.0 g, Yeast extract 5.0 g and NaCl 5.0 g for 1 liter, pH 7.2) and MM (Sucrose 5.0 g, KH_2_PO_4_ 2.0 g, (NH_4_)_2_HPO_4_ 2.0 g, MgSO_4_·7H_2_O 1.0 g, FeSO_4_·7H_2_O 0.01 g and MnSO_4_ 0.01 g for 1 liter, pH 7.2), were employed in the assay. Two kinds of compound concentrations, 1 mM and 10 mM, were tested respectively. The OD_600_ value of bacterial cultures at different time points was monitored by a Nanodrop 2000 spectrophotometer.

## Additional Information

**How to cite this article**: chen, L. *et al*. Ornithine Transcarbamylase ArgK Plays a Dual role for the Self-defense of Phaseolotoxin Producing *Pseudomonas syringae* pv. *phaseolicola*. *Sci. Rep.* **5**, 12892; doi: 10.1038/srep12892 (2015).

## Supplementary Material

Supplementary Information
