# Supplementary material for: Ornithine Transcarbamylase ArgK Plays a Dual role for the Self-defense of Phaseolotoxin Producing Pseudomonas syringae pv. phaseolicola
Source: Sci Rep. 2015 Aug 10;5:12892. doi: 10.1038/srep12892 (PMC4530439; doi:10.1038/srep12892)
Supplement: Supplementary Information [file srep12892-s1.pdf]

Supplementary Information

Ornithine Transcarbamylase ArgK Plays a Dual role for the Self-defense of Phaseolotoxin Producing *Pseudomonas syringae* pv. *phaseolicola*

Li CHEN, Pin LI, Zixin DENG, Changming ZHAO*

Key Laboratory of Combinatorial Biosynthesis and Drug Discovery, Ministry of Education, and School of Pharmaceutical Sciences, Wuhan University, Wuhan, 430071, China.

* Correspondence: cmzhao03@whu.edu.cn (CZ)

Running Title: ArgK's role in PHT producers' self-defense

Table S1 PCR Primers used in this study

Primer name	Primer sequence
DUF1	GCTTTTATCAGAAAAATGAAAGCTAATTTGAATTGGGGAGTCATTCCGGGGATCCGTCGACC
DUR2	AGGAATTTCAAAGCTTCCTCACACCTGAATGTTAAGAGTGATTGTAGGCTGGAGCTGCTTC
VUF1	TTACGCGATGTGCAGTAGGATA
VUR2	AATGGCGATACCTGACACCT AT
DQF1	GATCTGGTTATTTTTGAATATTGAATTTACGAGAGGCAGTTCATTCCGGGGATCCGTCGACC
DQR2	ATGAACACTCGGAGGGCGCTAGGTATGCCTGCGGCTGCCCATTGTAGGCTGGAGCTGCTTC
VQF1	CGCAGGGTTTGAGCCGATTCTA
VQR2	AGTCGCCATTACTCCGTACACG
DLF1	TGGGTTCTTGTGCCGCCAATTTAAAGGAAATTAACCGGTGATTCCGGGGATCCGTCGACC
DLR2	TGCAAAGCTGATCTGAAAGGCATCGGCGAAACACTTTTCATGTAGGCTGGAGCTGCTTC
VLF1	TGGACCATTCAACTTTTGGCCGTCA
VLR2	GCGAATGAGATGAGGTATCTGGCAA
P_{phtA}F1	TAAGATTAGCGGATCCCATTAGTGTAGGCAGGGC
P_{phtA}R2	ACTTATTACTTCCTTTTTCGT
PhtUF1	AAGGAAGTAATAAGTATGACGAATATATTAATGG
PhtUR2	CGACTCTAGAGGATCCTTAGTATGAAAGAGATACA
PhtQF1	AAGGAAGTAATAAGTGTGCCAATGAAAAAAATAATAT
PhtQR2	CGACTCTAGAGGATCCTCAAAGTGGTTCAGAACTA
OKF1	CGCGGATCCATGAAGATTACAAGCCTGAAAAACC
OKR2	CCGCTCGAGTCAGGGGACGACTGTCTCCAGCATC

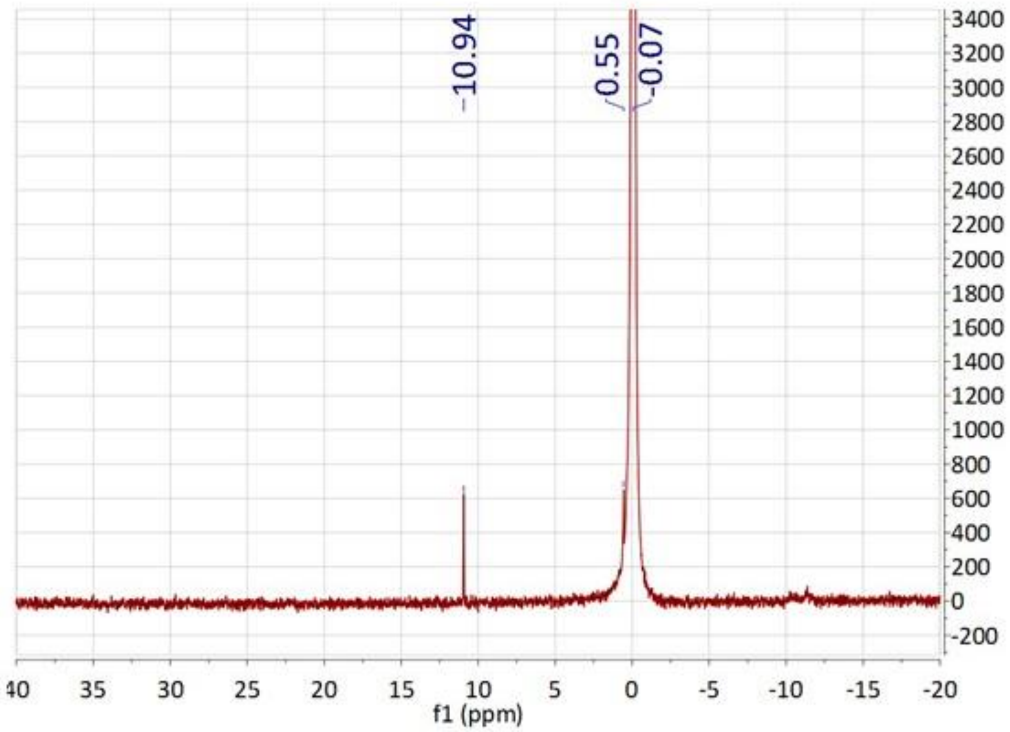

Figure S1 ^{31}P NMR spectrum of PHTs produced by *Pseudomonas syringae* pv. *phaseolicola* 1448A.

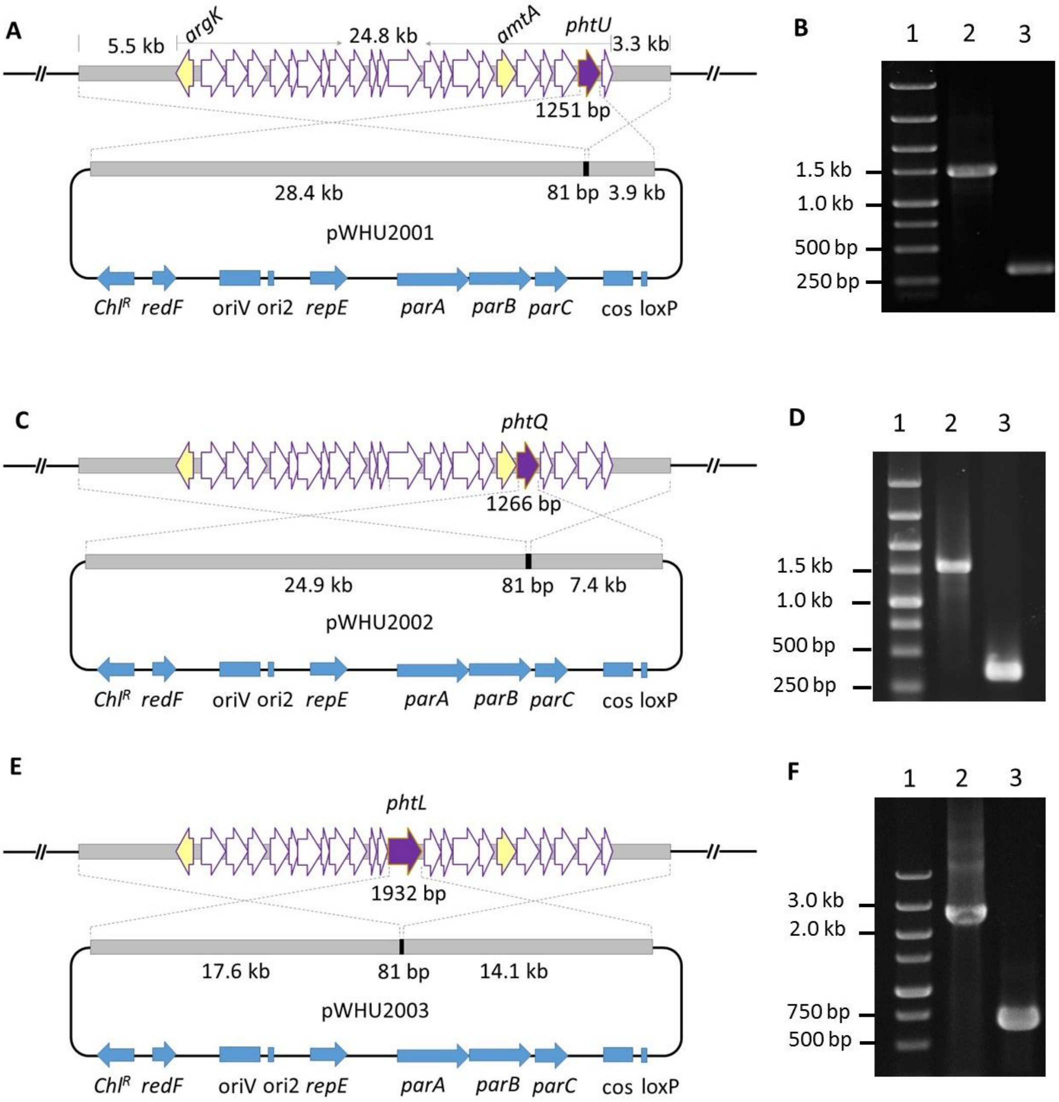

Figure S2 In-frame deletion of PHT biosynthesis related gene *phtU* (A), *phtQ* (C) and *phtL* (E), and PCR validation of *phtU*$^{-}$ (B), *phtQ*$^{-}$ (D) and *phtL*$^{-}$ (F) mutants. B, lane 1, DNA marker; lane 2, wild type (1471 bp); lane 3, *phtU*$^{-}$ mutant (301 bp). D, lane 1, DNA marker; lane 2, wild type (1531 bp); lane 3, *phtQ*$^{-}$ mutant (265 bp). F, lane 1, DNA marker; lane 2, wild type (2499 bp); lane 3, *phtL*$^{-}$ mutant (648 bp).

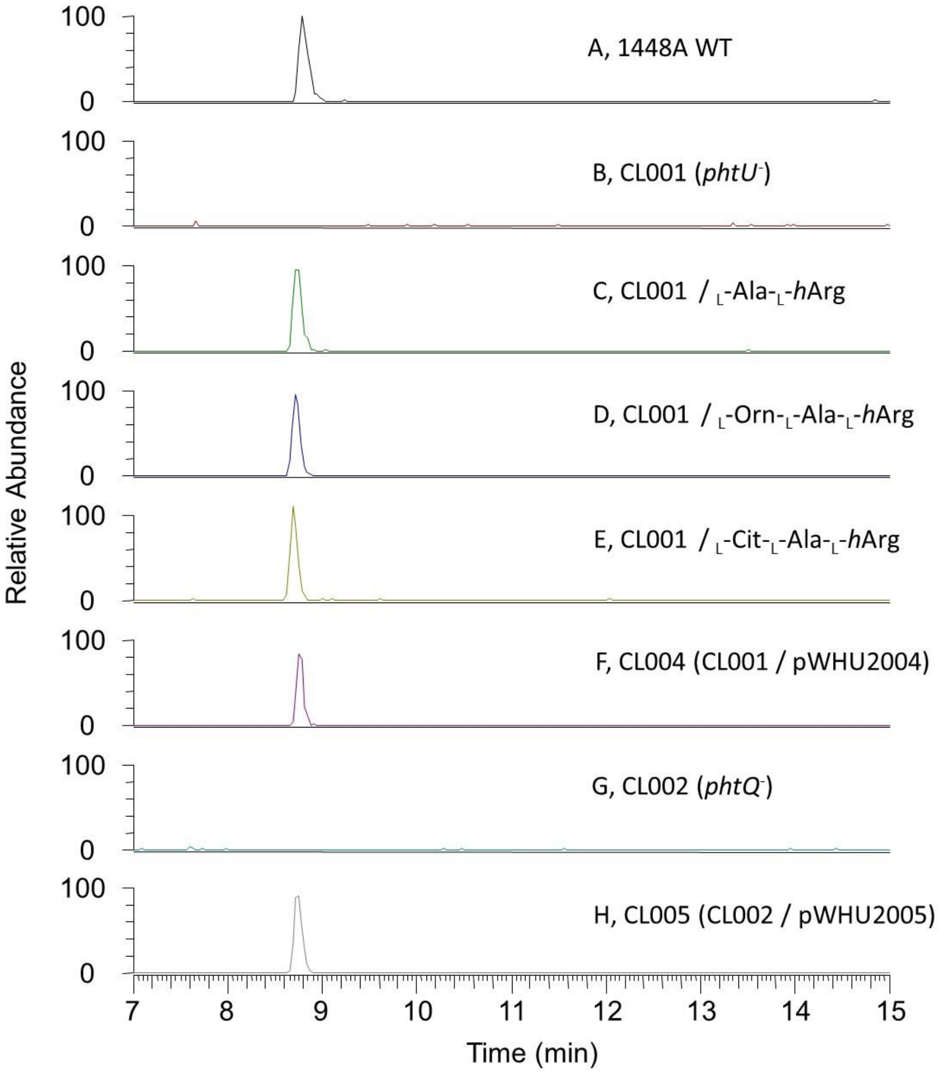

Figure S3 Extracted ion chromatograms of PHTs from culture supernatants of gene in-frame deletion mutants. Extracted with a tolerance of 0.5 Da. A, 1448A (PHT producer, wild type); B, *phtU*$^-$ mutant strain CL001; C, *phtU*$^-$ mutant strain CL001 fed with L-Ala -L-*h*Arg; D, *phtU*$^-$ mutant strain CL001 fed with L-Orn- L-Ala- L-*h*Arg; E, *phtU*$^-$ mutant strain CL001 fed with L-Cit- L-Ala- L-*h*Arg; F, *phtU*$^-$ mutant gene complementation strain CL004, with infusion gene P_{phtA}-ORF_{phtU} in pWHU2004; G, *phtQ*$^-$ mutant strain CL002; H, *phtQ*$^-$ mutant gene complementation strain CL005, with infusion gene P_{phtA}-ORF_{phtQ} in pWHU2005.

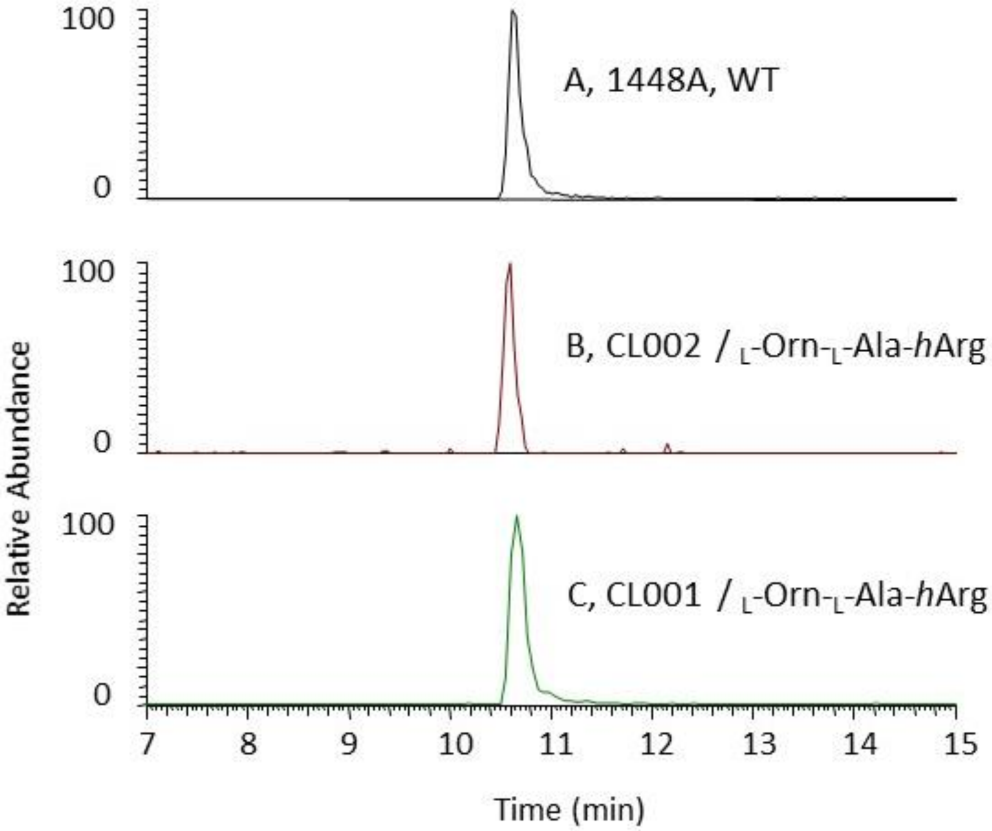

Figure S4 Extracted ion chromatograms of L-Cit- L-Ala- L-*h*Arg from mutant strains culture supernatants. Extracted with a tolerance of 0.5 Da. A, 1448A (PHT producer, wild type); B, *phtQ*$^-$ mutant strain CL002 fed with L-Orn- L-Ala- L-*h*Arg as substrate; C, *phtU*$^-$ mutant strain CL001 fed with L-Orn- L-Ala- L-*h*Arg as substrate.

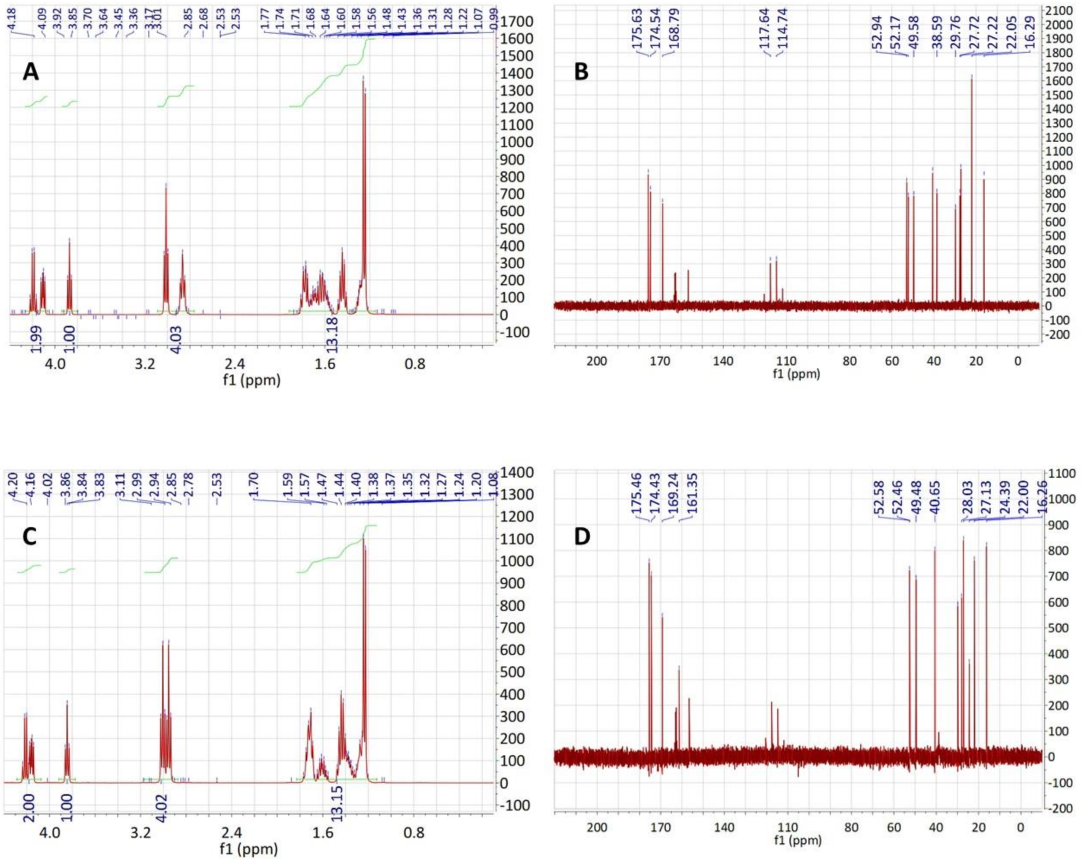

Figure S5 ^{1}H spectra of tripeptide standards L-Orn-L-Ala-L-*h*Arg (A) and L-Cit-L-Ala-L-*h*Arg (C) and ^{13}C NMR spectra of L-Orn-L-Ala-L-*h*Arg (B) and L-Cit-L-Ala-L-*h*Arg (D).
